# Infectious disease pandemic planning and response: Incorporating decision analysis

**DOI:** 10.1371/journal.pmed.1003018

**Published:** 2020-01-09

**Authors:** Freya M. Shearer, Robert Moss, Jodie McVernon, Joshua V. Ross, James M. McCaw

**Affiliations:** 1 Modelling and Simulation Unit, Centre for Epidemiology and Biostatistics, Melbourne School of Population and Global Health, The University of Melbourne, Melbourne, Australia; 2 Peter Doherty Institute for Infection and Immunity, The Royal Melbourne Hospital and The University of Melbourne, Australia; 3 Murdoch Children’s Research Institute, The Royal Children’s Hospital, Melbourne, Australia; 4 School of Mathematical Sciences, The University of Adelaide, Adelaide, Australia; 5 School of Mathematics and Statistics, The University of Melbourne, Melbourne, Australia

## Abstract

Freya Shearer and co-authors discuss the use of decision analysis in planning for infectious disease pandemics.

**Summary points**

- Planning is critical to mitigating the sudden and potentially catastrophic impact of an infectious disease pandemic on society. National pandemic policy documents cover a wide variety of control options, often with nonspecific recommendations for action.
- Despite advances in analytical methods for gaining early situational awareness (i.e., of a disease’s transmissibility and severity) and for predicting the likely effectiveness of interventions, a major gap exists globally in terms of integrating these outputs with the advice contained in policy documents.
- Decision models (and decision science as a field, more broadly) provide an approach to defining and evaluating alternative policy options under complex and changing conditions.
- A decision model for infectious disease pandemics is an appropriate method for integrating evidence from situational and intervention analysis tools, along with the information in policy documents, to provide robust advice on possible response options (including uncertainty).
- A decision model for pandemic response cannot capture all of the social, political, and ethical considerations that impact decision-making. Such a model should therefore be embedded in a decision support system that emphasizes this broader context.

## Introduction

Planning is critical to mitigating the sudden and potentially catastrophic impact of an infectious disease pandemic on society, but it is far from straightforward [1]. During a pandemic, decisions will be made under rapidly changing, uncertain conditions, with limited (if any) prior experience.

The 1918 H1N1 pandemic was estimated to have caused the death of tens of millions of people worldwide. It is encouraging that antivirals and vaccines available to us today would help to reduce the impact of a similar pandemic event, yet with cities and countries increasingly connected by air travel, we will likely be faced with a pathogen capable of spreading rapidly across the globe. The 2009 pandemic H1N1 (A(H1N1)pdm09), a virus estimated to be less transmissible than the 1918 strain [2], spread to 74 countries within just 4 months [3].

Mathematical and statistical models are important tools for pandemic planning and response. Although it is unlikely that we will ever be able to predict precisely where or when the next pandemic will occur [4], once an outbreak of pandemic potential has been identified, models have enormous potential to improve the effectiveness of our response. They can be used to synthesize the available data to provide enhanced situational awareness, to predict the future course of the pandemic and likely associated social and economic costs, and to plan mitigation strategies [5, 6].

## The role of modeling in current pandemic response policy

### Pandemic modeling trends

Modeling is a well-established approach to improving pandemic preparedness and response capabilities. In 1973, Fox and colleagues described the use of pandemic simulation models based on pathogen characteristics akin to 1957 H2N2 and 1968 H3N2 to explore the potential impact of mass vaccination and school closures [7, 8].

Decades later, modelers and policy makers employed similar methods in responding to influenza A(H1N1)pdm09. By leveraging surveillance systems and computational power not available to their predecessors in 1968, a variety of models were developed to provide real-time assessments of the pandemic impact level [9, 10] and effectiveness of possible control measures [10]. Additionally, many assumptions contained within the policy documents used in 2009 were based on prepandemic models [6, 11–14], and since 2009, models have increasingly supported the revision (and creation) of pandemic plans [15–17]. In recent decades, other global infectious disease events, including the epidemics of severe acute respiratory syndrome (SARS, 2002–2003), the emergence of highly pathogenic avian influenza (HPAI) virus H5N1 (2003), and the west African Ebola virus disease epidemic (2013–2016), have also stimulated advances in pandemic preparedness and response capabilities [11, 18, 19].

The pandemic preparedness and response models produced from these efforts can be broadly classified into two groups: those aiming to inform situational awareness and those aiming to understand the merits of possible interventions.

### The importance of situational awareness

A key lesson from the emergence of influenza A(H1N1)pdm09 was the need for pandemic policies to be adaptable to evolving pandemic scenarios [20, 21]. Many countries found that their planning assumptions did not match the expected level of pandemic impact because they were based on the more lethal HPAI H5N1 virus [22, 23]. In light of the relative mildness of A(H1N1)pdm09, which still had serious consequences, countries had to rapidly adjust their plans in order to deliver a proportionate response [20].

The World Health Organization (WHO) guiding document for pandemic influenza preparedness and response has since adopted a more flexible approach, emphasizing the importance of actions that can be scaled and targeted as needed [24], and this has been reflected in updated country plans [25–27]. In the current generation of pandemic plans, pandemic impact is typically considered in terms of disease transmissibility and severity [25, 27, 28]. Transmissibility describes how effectively the disease transmits between people. It strongly influences how quickly the epidemic grows, when it peaks, its overall magnitude, and how long it lasts. Severity determines how many people will become seriously unwell or die as a result of the disease.

At the onset of a pandemic, these pathogen characteristics will be unknown and must therefore be characterized as they emerge, because even pandemics of well-characterized pathogens will differ in these measures sufficiently to create uncertainty as to the best response. As our understanding of the probable impact of a pandemic improves, policy makers can then use this information to help decide on the overall scale of response, which control measures to implement, and when to deploy them [29]. Given the dependency of response plans and decision-making on assessments of situational awareness, gathering the appropriate information as early as possible in an outbreak has been identified as a priority for surveillance and real-time data analysis activities [30, 31].

To this end, advances have recently been made in the design of early outbreak surveillance methods such as First Few Hundred (FF100) household transmission studies [26] and the development of novel algorithms for analyzing the resulting data [32]. FF100 studies involve the collection of data from confirmed infections and their household contacts, including the date of symptom onset and final outcome, until a satisfactory characterization of the pathogen is achieved [26]. The use of these protocols is recommended as part of enhanced early surveillance activities in the current pandemic plans of the United Kingdom [26] and Australia [27], and WHO recommends a detailed investigation of at least the first 100 confirmed cases of any nascent pandemic [33]. These rapid, enhanced surveillance activities can be resource intensive but provide rich epidemiological data and overcome many quality, timeliness, and bias issues often associated with routine surveillance practices [29]. Further, when these data are analyzed with FF100-specific algorithms [32, 34], estimates of pathogen transmissibility and severity are obtained, enabling timely identification of the pandemic scenario that best characterizes an actual outbreak.

Similarly, epidemic forecasting algorithms that leverage routine surveillance data can also be used to rapidly predict pandemic characteristics relevant to policy makers. Every year during the influenza season, modelers in many parts of the world, sometimes in collaboration with public health practitioners, make weekly forecasts of epidemic characteristics, such as peak size and timing [35–37]. Since 2013, the United States Centers for Disease Control and Prevention (CDC) have even coordinated seasonal challenges to external researchers to predict onset week and peak week for the US influenza season [38]. Real-time forecasting has also been used to enhance situational awareness in outbreaks of other diseases of public health interest, including the west African Ebola virus disease epidemic (2013–2016) [19]. The Research and Policy for Infectious Disease Dynamics (RAPIDD) program subsequently hosted an Ebola forecasting challenge involving teams of modelers from both academic institutions and government agencies, with the goal of using “peace-time” to assess model performance and improve coordination between modeling groups [39].

### Assessing the response options

Once there are estimates of the transmissibility and severity of a pathogen, policy makers can use this information to decide how to respond. These decisions are often informed by the results of intervention modeling analyses. These analyses are either conducted during preparedness planning, with (static) outcomes embedded in policy documents, or they are developed in real time as part of emergency response. Intervention modeling involves simulating an epidemic in a population, with and without the intervention of interest, and comparing the outcomes [7, 15–17, 40–42]. These modeling studies have suggested that specific interventions are only effective under certain circumstances [15–17, 40, 41]. For example, in pandemic influenza scenarios in which clinical symptoms are severe (and thus highly visible to the healthcare system) and transmissibility is low, simulations suggest that liberal distribution of antivirals may completely avert the pandemic. On the other hand, if a pandemic virus exhibits low clinical severity and high transmissibility, antivirals alone would not be effective at reducing transmission or the burden on healthcare settings, and their primary utility will stem from their direct clinical benefits [28].

## A decision support system for pandemic response

Despite advances in methods for gaining situational awareness and assessing intervention impact, a major gap exists in terms of integrating the outputs from these methods with the advice contained in pandemic response policy. Policy documents will typically recognize the importance of methods for estimating pandemic impact (such as FF100), and their response advice is often informed by intervention models, but they do not articulate how these data and analytics will contribute to decision-making in real time during a pandemic.

The need for data collection and analysis pipelines to be made routine in epidemic response practice has been the topic of recent widespread discussion [30, 31, 43, 44]. Furthermore, it is clear that situational evidence should be used with intervention models to assess the likely effectiveness of response options. Our contribution to this evolving discussion is to highlight the need for formalizing—and exercising—precisely how emerging evidence is synthesized and used to support the decision-making processes articulated in policy documents, as part of preparedness activities.

Drawing on established practice from the discipline of decision science, we argue that a decision model is required to partly address this implementation gap—one that combines evidence from situational awareness tools and intervention models, along with the information in response policy, to evaluate alternative response strategies. This is realized in a statistical framework to appropriately capture and propagate uncertainties throughout the inference and evaluation processes. Such a decision model would provide robust recommendations on response options, including advice on uncertainty with respect to future epidemic behavior and likely effectiveness of alternative response strategies. We further argue that the decision model should be embedded in a broader decision support system that formally incorporates other information relevant to the decision-making process, including stakeholder values, taking us well beyond any current-generation planning and response capabilities.

This approach to decision-making has been applied in other settings in which decisions must be made in real time, under conditions of high complexity or uncertainty, including aviation [45], engineering [46], wildfire management [47], and livestock disease control [48–51]. In the context of human disease, although some have considered how to optimize interventions given dynamic knowledge of a system (including emerging epidemic data and resource availability), they tend to ignore the broader context in which decisions are made [52].

**Fig 1:**
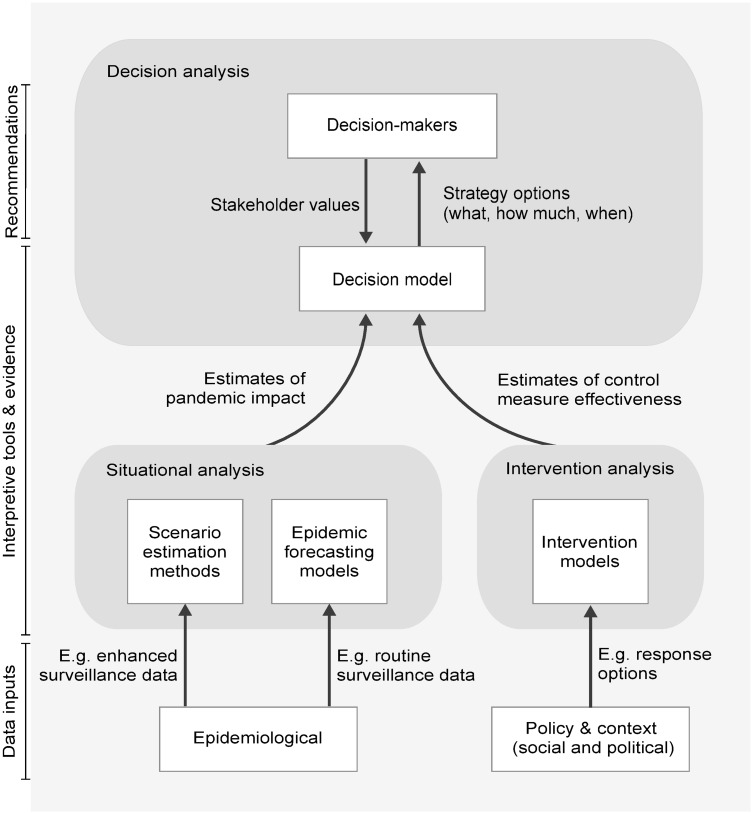
Proposed decision support system. Schematic of a proposed decision support system for infectious disease pandemic response.

Fig 1 depicts a proposed decision support system for pandemic response, featuring a statistical decision model that combines dynamic information from situational awareness tools and intervention models, along with the static information in response plans, and provides dynamic advice on optimal response strategies. When operating, the system would continually update as information becomes available, enabling decision-makers to revise and refine control measures over time, including making difficult decisions about scaling back or ceasing an intervention activity.

Our ideas build on the decision-making framework developed by Lipsitch and colleagues [29], which defines the data and interpretive tools required for a pandemic response in terms of the key public health decisions that must be made. Although they discuss an “idealized” progression from epidemiological and surveillance data to evidence and then to evidence-based decisions, they also acknowledge that other sources of data and evidence should and do influence decision-making. We have extended their framework by adding policy and contextual data and stakeholder priorities as inputs, as well as an additional layer of evidence interpretation—the decision model—which offers specific strategies (what, how much, when) to decision-makers.

### Case study: Antiviral decision model for pandemic influenza in the Australian context

**Fig 2:**
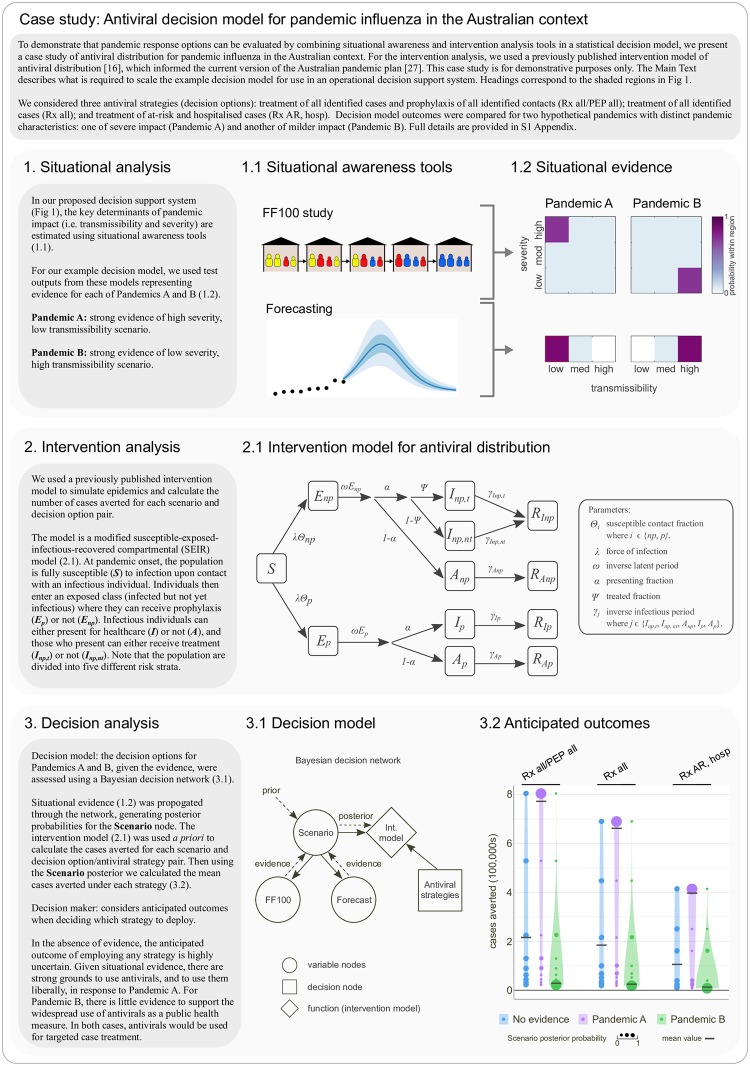
Case study. Antiviral decision model for pandemic influenza in the Australian context. FF100, First Few Hundred.

In order to demonstrate that outputs from situational and intervention analyses, when combined using a statistical decision model, can provide recommendations on response options (including uncertainty), we present a realistic example of an antiviral decision problem for pandemic influenza in Fig 2 (full details are provided in S1 Appendix). For the intervention analysis component, we have used our previously published intervention model of targeted antiviral distribution strategies [16]. This model and its findings form the basis for Australia’s current pandemic response plan [27, 53]. The model allows for the use of antivirals for treatment of cases and postexposure prophylaxis of contacts, differential risks of severe disease outcomes and differential benefits of treatment across population subgroups, and health system capacity constraints. The most recent version of this model is described by Moss and colleagues [16], and it builds on a larger body of work, conducted over a 15-year period, which has focused on developing pandemic antiviral policy for the Australian context [13–17, 28, 54].

In presenting this example, we have necessarily and deliberately kept the decision model to a minimum working example in order to focus on the broader decision analysis aspects of the problem and on the types and flow of information required by the model. As such, the decision model is limited to a single intervention (antivirals), with each strategy implemented at the start of the response phase (as defined by the Australian pandemic plan) for the remaining duration of the pandemic or until antiviral stockpile depletion. A decision model within a fully operational system would, of course, require a much higher dimensional decision space, including the use of an intervention model incorporating multiple interventions and the ability to integrate over all feasible intervention start and stop times. It would also require consideration of the computational implementation of the decision model to ensure timely (possibly daily) availability of situation-specific intervention model outcomes that captures uncertainty in FF100 estimates of epidemiological parameters (i.e., severity and transmissibility), as well as intervention parameters (e.g., drug effectiveness) and operational parameters (e.g., daily antiviral distribution capacity). It may also be important to reconcile potentially distinct transmission models used for the inference of disease characteristics and the assessment of interventions.

### The decision context

Although there is clearly much further technical work to do, these aspects are perhaps the most straightforward part of developing an operational system; more challenging is working with stakeholders to decide on the structures and outcomes of each component and how different types of evidence should be weighted. This depends on the social and political context in which decisions are made. For example, the availability and acceptability of interventions will depend on a host of social and political factors, which may change as the pandemic progresses [55–58]. Jurisdictional and community values must be carefully elicited and incorporated into the decision support system, not least because we know that pandemic response policies have the potential to perpetuate and exacerbate existing social disparities [59]. As shown in Fig 1, certain decision-maker priorities can be incorporated in system design (such as whether one type of evidence is more trusted than another), but ultimately, it is not expected that all social, political, and ethical considerations will be captured by system structures or parameters.

Decision science can contribute to pandemic preparedness and response not only by providing analytical tools for evaluating response options but also by providing a structured and inclusive approach for incorporating these tools into decision-making [60]. This approach includes formally engaging with decision-makers to clearly define their response objectives and to design and agree on suitable metrics for assessing alternative response strategies. The role of the decision support system would be not to produce a single optimal strategy but to clearly and transparently present decision options in a way that effectively helps decision-makers choose the strategy most aligned with achieving their objectives. Examples of this approach exist in conservation [61, 62] and livestock disease management [49, 50]. Further, Moss and colleagues [35, 63] describe their collaborative engagement with public health decision-makers in model development for seasonal influenza forecasting, which provides useful insight into their process and the value of these engagements.

## Discussion

Pandemic response capabilities will be improved by formally integrating outputs from situational and intervention analyses with pandemic response policy. We have proposed one such approach to doing so—a decision model embedded within a broader decision support system that recognizes the social and political context in which decisions are made. Under this approach, we draw on well-established analytical tools used in the discipline of decision science (that is, decision models) and argue that the broader decision support system should be developed using decision science principles.

A system developed using this approach will ensure that the most complete, robust information is available to decision-makers at operationally relevant time points. For example, such a system will enable the development of methods (that simultaneously account for relevant sources of uncertainty) for triggering key policy decisions, such as determining when to switch from general response strategies (when knowledge is scarce) to more proportionate and targeted response strategies (when sufficient knowledge is gained). This switch has significant resource implications because it signals the sufficient acquisition of FF100 data and the cessation of resource-intensive FF100 studies.

The testing and evaluation of our proposed system is an important challenge for its operational use. In order to evaluate the system against actual situational evidence from FF100 and forecasting, rather than the hypothetical evidence used in Fig 2, we would require the relevant data to be collected concurrently during an outbreak. An initial evaluation step could involve conducting an FF100 trial during a seasonal influenza epidemic in a jurisdiction where seasonal forecasting tools are already routinely used. In addition to providing data against which to evaluate the performance of algorithms and models within the system, this would enable the identification of operational challenges associated with the FF100 study design and its implementation. Tabletop exercises would also be important for testing and improving the system, particularly to obtain feedback on the clarity of presentation of alternative response strategies and uncertainties. Tabletop exercises/response drills are already a matter of routine in many jurisdictions; we are calling for analytics to be an integral part of these exercises.

Although we have focused on the effective use of antivirals in an influenza pandemic in the decision model example, our ideas are relevant and adaptable to other diseases of pandemic/epidemic potential. It will be important to next incorporate a suite of nonspecific interventions, such as social distancing, border screening, and infection control measures, which are effective against a broader range of infectious diseases. FF100 data collection protocols and algorithms are adaptable to diseases other than influenza, and outbreaks of emerging pathogens such as SARS, for which pharmaceutical interventions were not available, have stimulated modeling research into the control of such pathogens. This has resulted in further development of intervention models for nonspecific control measures, including isolation and quarantine [64–66].

Under conditions of high stress and uncertainty, a pandemic response is more likely to succeed if responders have access to key information in a timely and coherent manner. Formal integration of outputs from situational awareness and intervention analysis methods with the information contained within policy documents will improve the ability of decision-makers to assess their response options in a given pandemic event. We have demonstrated a novel method for doing so (Fig 2) and illustrated how it would fit into a broader decision support system (Fig 1). We argue that such a system will best support the making of robust and transparent decisions when developed through a decision science process, emphasizing the social and political needs of pandemic planning efforts [60].

Drawing on our example decision model (Fig 2) and a host of published examples of stakeholder engagement in decision-making processes [61, 62, 67], we suggest that both the technical and nontechnical challenges associated with developing a decision support system are surmountable. Having such a system in place—and articulated in pandemic policy documents—will be of great value to decision-makers when the next pandemic inevitably arrives.

## Supporting information

S1 Appendix Methods supplement to Fig 2. (PDF)

## Abbreviations

CDC: Centers for Disease Control and Prevention
FF100: First Few Hundred
HPAI: highly pathogenic avian influenza
RAPIDD: Research and Policy for Infectious Disease Dynamics
SARS: severe acute respiratory syndrome
WHO: World Health Organization
