# Supplementary material for: Infectious disease pandemic planning and response: Incorporating decision analysis
Source: PLoS Med. 2020 Jan 9;17(1):e1003018. doi: 10.1371/journal.pmed.1003018 (PMC6952100; doi:10.1371/journal.pmed.1003018)
Supplement: S1 Appendix — (PDF) [file pmed.1003018.s001.pdf]

S1 Appendix – Methods supplement to Fig 2 of Infectious disease pandemic planning and response: incorporating decision analysis

Freya M. Shearer[1], Robert Moss[1], Jodie McVernon[1,2,3], Joshua V. Ross[4], and James M. McCaw[1,2,5]

1. Melbourne School of Population and Global Health, The University of Melbourne, Australia
2. Peter Doherty Institute for Infection and Immunity, The Royal Melbourne Hospital and The University of Melbourne, Australia
3. Murdoch Childrens Research Institute, The Royal Children's Hospital, Australia
4. School of Mathematical Sciences, The University of Adelaide, Australia
5. School of Mathematics and Statistics, The University of Melbourne, Australia

Overview

This Appendix supports the example decision model presented in Fig 2 of the manuscript "Case study: Antiviral decision model for pandemic influenza in the Australian context".

The purpose of this case study is to show that outputs from situational and intervention analyses, when combined using a statistical decision model (specifically a Bayesian decision network), can provide recommendations on response options (including uncertainty). For the intervention analysis, we used our previously published intervention model of targeted antiviral distribution strategies [1]. This model and its findings form the basis for Australia's current response plan [2]. The model allows for: the use of antivirals for treatment of cases and post-exposure prophylaxis of contacts; differential risks of severe disease outcomes and differential benefits of treatment across population subgroups; and health system capacity constraints. The most recent version of this model is described by Moss and colleagues [1] and it builds on a larger body of work, conducted over a 15-year period, which has focused on developing pandemic antiviral policy for the Australian context [1, 3, 4, 5, 6, 7, 8].

We considered three different antiviral strategies (decision options): treatment of all identified cases and post-exposure prophylaxis of all identified contacts (Rx all/PEP all); treatment of all identified cases (Rx all); and treatment of at-risk and hospitalised cases (Rx AR, hosp). Decision model outcomes were compared for two hypothetical pandemics with distinct pandemic characteristics: one of severe impact (Pandemic A) and another of milder impact (Pandemic B) (see Section 2). The decision options for Pandemics A and B, given the evidence, were assessed using a Bayesian decision network.

It is important to note that the decision model presented in this case study is for demonstrative purposes only. The main text of the manuscript describes what is required to scale this example decision model for use in a fully operational decision support system.

S1 Appendix begins with a background section on Bayesian networks, and then steps through each section of Manuscript Fig 2.

1 Background on Bayesian networks

Bayesian networks are a powerful tool for reasoning under uncertainty. They provide a graphical (and thus intuitive, visual and explicit) representation of relationships between components of complex systems, can manage incomplete domain knowledge, and are able to incorporate inputs from multiple scales and formats, including expert opinion [9, 10]. Bayesian networks have been widely applied in many disciplines, and have previously been used to support clinical and public health decision-making including by assisting in disease diagnosis [11, 12], forecasting of epidemic spread [13], and detecting outbreaks of rare pathogens [14].

A Bayesian network is represented by a graph, composed of a set of nodes connected by arrows, known as a *directed acyclic graph*. Nodes represent random variables and arrows indicate probabilistic relationships among nodes. Arrows terminate at 'child' nodes, denoted X, and originate at parent nodes, denoted $Pt(X)$. A parent node can also be a 'root' node. Each node/variable (X) can take a value, and has an associated conditional probability table (for discrete variables), or distribution (for continuous variables) – we denote these as CPT or CPD respectively. The CPT or CPD contains the probability of the child node taking any particular value, given the value of its' parents (*i.e.*, $P(X \mid Pt(X))$). For root nodes – *i.e.*, nodes without any parents (*i.e.*, $Pt(X) = \emptyset$) – the CPT or CPD contains the prior probability for the node.

The full joint probability distribution is given by the product of all the probability distributions (prior and conditional) in the network, as shown in Equation 1 below.

$$P(X_1, ..., X_n) = \prod_{i=1}^{n} P(X_i | Pt(X_i)) \quad (1)$$

Thus, a Bayesian network is a compact factorisation of the joint probability distribution P over random variables $X_1, ..., X_n$. From the joint distribution we can compute a number of probabilities of interest. The structure and parameters (prior and conditional probabilities) of a Bayesian network can be either manually elicited from expert opinion or estimated from data [10, 9].

Standard Bayesian networks can be extended for decision-making by adding decision and utility variables that explicitly represent possible decisions and the utilities of possible outcomes [9].

In the context of providing decision-support for pandemic influenza, a Bayesian decision network can probabilistically combine information from situational and intervention analyses to provide guidance to decision-makers on whether or not to implement a given control option.

2 Situational analysis (Section 1 of Manuscript Fig 2)

In our proposed decision support system (see Manuscript Fig 1), the key determinants of pandemic impact (*i.e.*, transmissibility and severity) are assessed using situational awareness tools [15, 16]. For this case study, both characteristics are considered using a 'Low', 'Moderate', 'High' scale, resulting in nine possible pandemic scenarios (*i.e.*, levels of impact), which is consistent with the scenarios defined within Australia's current pandemic influenza response plan.

For our example decision analysis, we used test FF100 and forecasting analysis outputs for Pandemics A and B. These outputs were designed to provide strong evidence of a high severity, low transmissibility scenario for Pandemic A, and strong evidence of a low severity, high transmissibility scenario for Pandemic B (Figure 1).

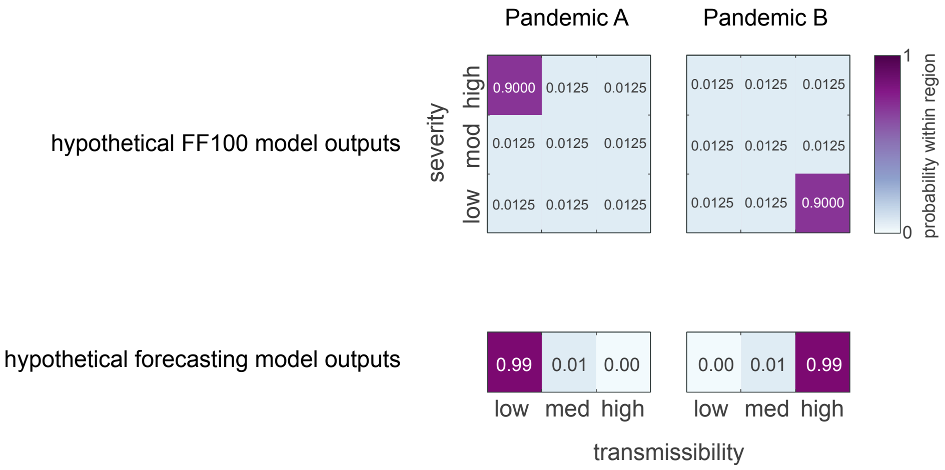

Figure 1: Situational evidence for Pandemics A and B from FF100 and forecasting analysis outputs.

3 Intervention analysis (Section 2 of Manuscript Fig 2)

The outcome of a decision depends on both the pandemic scenario and the action taken (*i.e.* which antiviral strategy is deployed).

We used our previously published intervention model of targeted antiviral distribution [1] to simulate epidemics and calculate the total number of cases for each scenario and decision option pair.

3.1 Intervention model for targeted antiviral distribution (Section 2.1 of Manuscript Fig 2)

A full description of the intervention model and each of the targeted antiviral distribution strategies can be found in [1]. Briefly, the model is based on a modified susceptible-exposed-infectious-recovered (SEIR) compartmental model. At pandemic onset, the population is fully susceptible S to infection upon contact with an infectious individual. Individuals then enter an exposed class (infected but not yet infectious) where they can receive prophylaxis (E_p) or not (E_{np}). Infectious individuals can either present for healthcare (I) or not (A), and those who present can either receive treatment ($I_{np,t}$) or not ($I_{np,nt}$). Once recovered from infection (R_{Inp}, R_{Anp}, R_{Ip}, R_{Ap}), individuals are assumed to be fully resistant to reinfection. The model also incorporates a dynamic "contact" label, applied to a fixed number of individuals drawn from the whole population each time a new infectious case appears. This allows simulation of targeted post-exposure antiviral prophylaxis (PEP). The Australian population was stratified into five distinct risk groups (young children, elderly, high-risk, health care workers, and the general adult population), to allow for differential risks of severe outcomes, differential benefits conferred by antiviral treatment, and targeted treatment and prophylaxis strategies. It was assumed that these groups mixed homogeneously. All simulations were performed as described by Moss and colleagues [1]. A schematic of the model structure is displayed in Figure 2.

3.2 Calculating the number of cases

For each of the nine pandemic scenarios and four antiviral distribution strategies (including no distribution), we performed 10,000 intervention model simulations, using Latin hypercude sampling (LHS) to account for model uncertainties (*e.g.*, epidemic time-course, effectiveness of antivirals). See Moss and colleagues [1] for further details on the LHS approach. We then calculated the mean number of cases (number of infectious individuals who present for health care) for each scenario and antiviral/decision option pair (see Table 4).

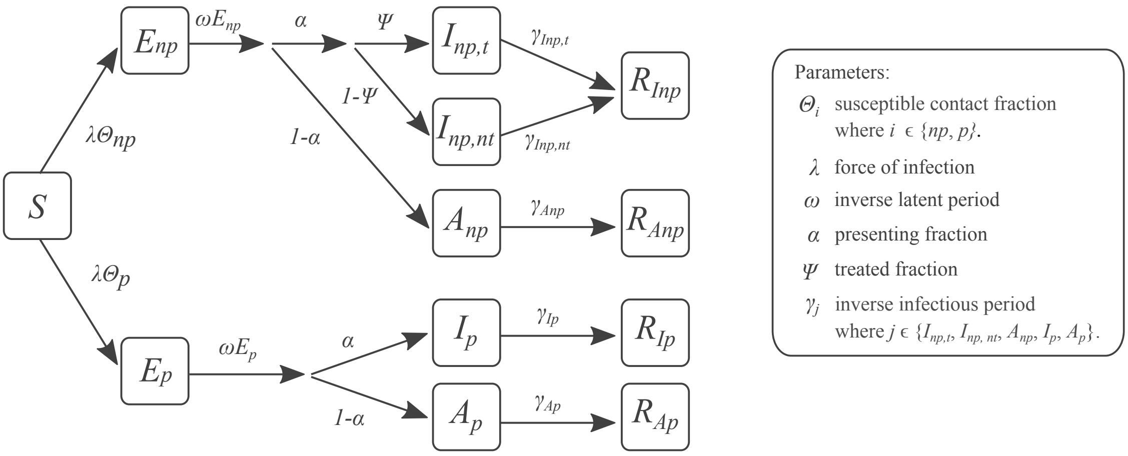

Figure 2: Schematic of the modified SEIR intervention model for targeted antiviral distribution.

4 Decision analysis (Section 3 of Manuscript Fig 2)

The decision options for Pandemics A and B, given the situational evidence, were assessed using a Bayesian decision network.

4.1 Bayesian decision network (Section 3.1 of Manuscript Fig 2)

The decision network described in 3.1 of Manuscript Fig 2 is shown in Figure 3. This network contains a single root node, `Scenario`, which is also the query node, representing the 'true' pandemic scenario. The `Scenario` node has nine possible states, representing nine possible pandemic scenarios categorised as either low, medium/moderate or high for both transmissibility and severity (*e.g.*, high severity, high transmissibility; high severity, medium transmissibility; high severity, low transmissibility *etc.*) [6]. `Scenario` is connected to two evidence nodes: `FF100`, representing an estimate of the pandemic scenario by the FF100 estimation algorithm (this node has the same nine states as `Scenario`), and `Forecast`, representing a forecasting model estimate of transmissibility (this node has three states: low, medium and high).

The network also contains a decision node `AVstrategies`, representing each of the different antiviral strategies (including inaction), and a function `IntModel` that calculates the number of cases. The arrows from `Scenario` and `AVstrategies` to `IntModel` indicate that the number of cases will depend on both the pandemic scenario (*i.e.,* transmissibility and severity) and the action taken (*i.e.,* which antiviral strategy is employed). `IntModel` is equivalent to the utility or value function described in the decision network literature, but for this example we chose to use the number of cases averted (compared to inaction) to evaluate the impact of each antiviral strategy. A description of each decision network node and their possible states can be found in Table 1.

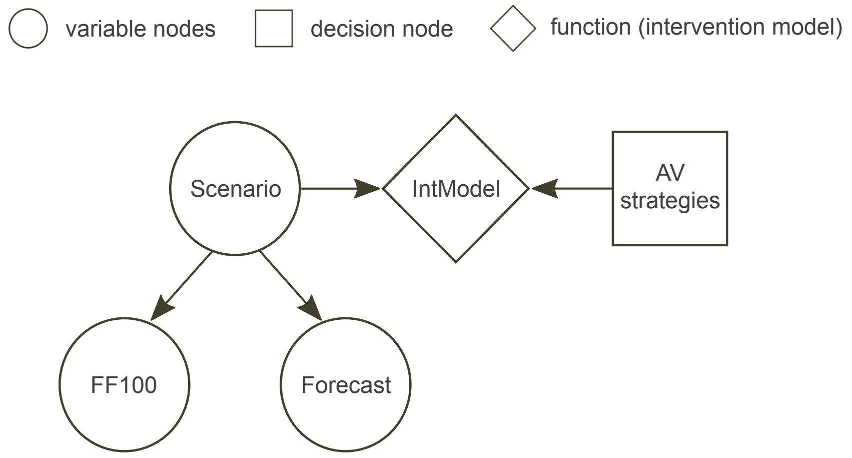

Figure 3: Schematic of the Bayesian decision network for pandemic influenza decision support.

Table 1: Description of decision network nodes, their type and their possible states.
*The `IntModel` function calculates the number of cases C for each scenario (of transmissibility $i \in \{low, medium, high\}$ and severity $j \in \{low, moderate, high\}$) and decision option $d \in \{Rxall/PEPall, Rxall, RxARhosp, Inaction\}$ pair.

Node	Type	States	Description
`Scenario`	variable (query)	low transmissibility, high severity<br>medium transmissibility, high severity<br>high transmissibility, high severity<br>low transmissibility, moderate severity<br>medium transmissibility, moderate severity<br>high transmissibility, moderate severity<br>low transmissibility, low severity<br>medium transmissibility, low severity<br>high transmissibility, low severity	'True' pandemic scenario
`FF100`	variable (evidence)	low transmissibility, high severity<br>medium transmissibility, high severity<br>high transmissibility, high severity<br>low transmissibility, moderate severity<br>medium transmissibility, moderate severity<br>high transmissibility, moderate severity<br>low transmissibility, low severity<br>medium transmissibility, low severity<br>high transmissibility, low severity	Estimated pandemic scenario
`Forecast`	variable (evidence)	low<br>medium<br>high	Estimated transmissibility
`IntModel`	function	*$C(i, j, d)$	Calculates the number of cases
`AVstrategies`	decision	Rx all/PEP all<br>Rx all<br>Rx AR, hosp	Antiviral strategies

4.2 Network conditional and prior probability tables

The CPTs for this network establish how much weight is given to estimates of the 'true' pandemic scenario arising from the different inputs, in this case the FF100 and forecasting analyses. We do not expect that either the FF100 or forecasting analyses will perfectly estimate the 'true' pandemic scenario, and this is reflected in the values that we assigned for the CPTs (see Tables 2 and 3). For example, even if 100% of the posterior samples for severity and transmissibility parameters from the FF100 model fall into the high severity, low transmissibility region, we nonetheless assigned the probability of `Scenario` being in the high severity, low transmissibility state to be 0.9, rather than 1. The prior probabilities of `Scenario`, which is a root node, were set to be uniform.

The values of the SEIR model parameters for each combination of the states of `Scenario` and `AVstrategy` nodes, as well as the calculated number of cases can be found in Table 4.

Table 2: Conditional probability table for `Forecast`, which has one parent, `Scenario`. t = transmissibility. s = severity. mod = moderate. med = medium.

	Scenario								
Forecast	high s low t	high s med t	high s high t	mod s low t	mod s med t	mod s high t	low s low t	low s med t	low s high t
high t	0.03	0.05	0.90	0.03	0.05	0.90	0.03	0.05	0.90
med t	0.07	0.90	0.07	0.07	0.90	0.07	0.07	0.90	0.07
low t	0.90	0.05	0.03	0.90	0.05	0.03	0.90	0.05	0.03

Table 3: Conditional probability table for `FF100`, which has one parent, `Scenario`. t = transmissibility. s = severity. mod = moderate. med = medium.

	Scenario								
FF100	high s low t	high s med t	high s high t	mod s low t	mod s med t	mod s high t	low s low t	low s med t	low s high t
high s, low t	0.9000	0.0125	0.0125	0.0125	0.0125	0.0125	0.0125	0.0125	0.0125
high s, med t	0.0125	0.9000	0.0125	0.0125	0.0125	0.0125	0.0125	0.0125	0.0125
high s, high t	0.0125	0.0125	0.9000	0.0125	0.0125	0.0125	0.0125	0.0125	0.0125
mod s, low t	0.0125	0.0125	0.0125	0.9000	0.0125	0.0125	0.0125	0.0125	0.0125
mod s, med t	0.0125	0.0125	0.0125	0.0125	0.9000	0.0125	0.0125	0.0125	0.0125
mod s, high t	0.0125	0.0125	0.0125	0.0125	0.0125	0.9000	0.0125	0.0125	0.0125
low s, low t	0.0125	0.0125	0.0125	0.0125	0.0125	0.0125	0.9000	0.0125	0.0125
low s, med t	0.0125	0.0125	0.0125	0.0125	0.0125	0.0125	0.0125	0.9000	0.0125
low s, high t	0.0125	0.0125	0.0125	0.0125	0.0125	0.0125	0.0125	0.0125	0.9000

4.3 Decision network evaluation

4.3.1 Evidence propagation

To evaluate the decision network, we first add any available evidence to the network, and then calculate the posterior probability of the query node. In our example, the query node is `Scenario` (since it is the only parent node of `IntModel`, aside from the decision node). This requires propagating evidence through the network, also known as belief updating, using a standard Bayesian network inference algorithm [9].

For our example model, situational evidence for Pandemics A and B (Figure 1) were in turn supplied to the `FF100` and `Forecast` nodes of the decision network. The posterior of the `Scenario` node, `Belief`, was then computed for Pandemics A and B using Kim and Pearl's 'message passing' algorithm in which messages, λ and π, are passed to the query node `query` from its child and parent nodes, respectively [17]:

$$\texttt{Belief}(i,j) = \alpha\lambda(i,j)\pi(i,j), \tag{2}$$

where α is a normalising constant rendering $\sum_{i,j}\texttt{Belief}(i,j) = 1$. $\lambda(i,j)$ is the message arriving at `query` from its children which is based on messages λ_c from each child c:

$$\lambda_{\texttt{query}}(i,j) = \prod_{c\in\texttt{children}}\sum_{k\in K_c}\lambda_c(k)P_c(k|i,j), \tag{3}$$

where P_c is from a fixed CPT relating child c to `query`, and `children` is the set of all child nodes and for each child c we define K_c to be the set states for child c. The equation for $\pi(i,j)$, which represents the message from parent nodes, is not described here since `Scenario` has no parent nodes.

In our example model, $\pi(i,j)$ is the prior distribution of `Scenario` and since we assume no prior knowledge of the pandemic scenario, $\pi(i,j) = 1/9$ for all i,j.

Further, in our example model we have $\texttt{children} \in (\texttt{FF100}, \texttt{Forecast})$ and for each child, $K_{\texttt{FF100}} \in ((1,1),(1,2),...,(3,3))$ and $K_{\texttt{Forecast}} \in (1,2,3)$. The message $\lambda_{\texttt{Scenario}}$ to the `Scenario` node combines information that has come from each child c, via messages λ_c, and the fixed CPT relating child c to `Scenario` (Tables 2 and 3). Messages λ_c are the evidence added at child node c, where $\lambda_{\texttt{FF100}}(m,n) = D_{\texttt{FF100}}[m,n]$ and $\lambda_{\texttt{Forecast}}(m) = D_{\texttt{Forecast}}[m]$ are evidence. For Pandemic A, the evidence is as follows (as per Figure 1):

$$D_{\texttt{FF100}}[m,n] = \begin{bmatrix} 0.9000 & 0.0125 & 0.0125 \\ 0.0125 & 0.0125 & 0.0125 \\ 0.0125 & 0.0125 & 0.0125 \end{bmatrix},$$

Table 4: Intervention model outcomes table. The following information is displayed: 1) Lower and upper values of transmissibility and severity used for intervention model simulations for each of the nine pandemic scenarios, which correspond to the nine states of the `Scenario` node. These values are taken from those shown in Table 2 of Moss and colleagues [1], R_0 for transmissibility and η for severity (the proportion of infections that, in the absence of early treatment, will require hospitalisation). 2) Each decision option/antiviral strategy. 3) The number of cases for each scenario and decision option pair, calculated as the mean of 10,000 intervention model simulations.

Transmissibility	R_0 lower	R_0 upper	Severity	η lower	η upper	`AVstrategy`	Cases
low	1.05	1.20	high	10^{-2}	10^{-1}	Rx all/PEP all	3.7×10^5
medium	1.20	1.40	high	10^{-2}	10^{-1}	Rx all/PEP all	1.9×10^6
high	1.40	1.70	high	10^{-2}	10^{-1}	Rx all/PEP all	3.3×10^6
low	1.05	1.20	moderate	10^{-3}	10^{-2}	Rx all/PEP all	3.4×10^5
medium	1.20	1.40	moderate	10^{-3}	10^{-2}	Rx all/PEP all	9.1×10^5
high	1.40	1.70	moderate	10^{-3}	10^{-2}	Rx all/PEP all	1.4×10^6
low	1.05	1.20	low	10^{-4}	10^{-3}	Rx all/PEP all	3.1×10^5
medium	1.20	1.40	low	10^{-4}	10^{-3}	Rx all/PEP all	7.8×10^5
high	1.40	1.70	low	10^{-4}	10^{-3}	Rx all/PEP all	1.2×10^6
low	1.05	1.20	high	10^{-2}	10^{-1}	Rx all	4.9×10^5
medium	1.20	1.40	high	10^{-2}	10^{-1}	Rx all	2.0×10^6
high	1.40	1.70	high	10^{-2}	10^{-1}	Rx all	3.3×10^6
low	1.05	1.20	moderate	10^{-3}	10^{-2}	Rx all	3.7×10^5
medium	1.20	1.40	moderate	10^{-3}	10^{-2}	Rx all	9.2×10^5
high	1.40	1.70	moderate	10^{-3}	10^{-2}	Rx all	1.4×10^6
low	1.05	1.20	low	10^{-4}	10^{-3}	Rx all	3.3×10^5
medium	1.20	1.40	low	10^{-4}	10^{-3}	Rx all	7.9×10^5
high	1.40	1.70	low	10^{-4}	10^{-3}	Rx all	1.2×10^6
low	1.05	1.20	high	10^{-2}	10^{-1}	Rx AR, hosp	7.6×10^5
medium	1.20	1.40	high	10^{-2}	10^{-1}	Rx AR, hosp	2.2×10^6
high	1.40	1.70	high	10^{-2}	10^{-1}	Rx AR, hosp	3.4×10^6
low	1.05	1.20	moderate	10^{-3}	10^{-2}	Rx AR, hosp	4.3×10^5
medium	1.20	1.40	moderate	10^{-3}	10^{-2}	Rx AR, hosp	9.5×10^5
high	1.40	1.70	moderate	10^{-3}	10^{-2}	Rx AR, hosp	1.4×10^6
low	1.05	1.20	low	10^{-4}	10^{-3}	Rx AR, hosp	3.8×10^5
medium	1.20	1.40	low	10^{-4}	10^{-3}	Rx AR, hosp	8.1×10^5
high	1.40	1.70	low	10^{-4}	10^{-3}	Rx AR, hosp	1.2×10^6
low	1.05	1.20	high	10^{-2}	10^{-1}	Inaction	1.2×10^6
medium	1.20	1.40	high	10^{-2}	10^{-1}	Inaction	2.4×10^6
high	1.40	1.70	high	10^{-2}	10^{-1}	Inaction	3.5×10^6
low	1.05	1.20	moderate	10^{-3}	10^{-2}	Inaction	4.7×10^5
medium	1.20	1.40	moderate	10^{-3}	10^{-2}	Inaction	9.8×10^5
high	1.40	1.70	moderate	10^{-3}	10^{-2}	Inaction	1.4×10^6
low	1.05	1.20	low	10^{-4}	10^{-3}	Inaction	4.0×10^5
medium	1.20	1.40	low	10^{-4}	10^{-3}	Inaction	8.2×10^5
high	1.40	1.70	low	10^{-4}	10^{-3}	Inaction	1.2×10^6

and

$$D_{\texttt{Forecast}}[m] = [0.99, 0.01, 0].$$

And for Pandemic B:

$$D_{\texttt{FF100}}[m,n] = \begin{bmatrix} 0.0125 & 0.0125 & 0.0125 \\ 0.0125 & 0.0125 & 0.0125 \\ 0.0125 & 0.0125 & 0.9000 \end{bmatrix},$$

and

$$D_{\texttt{Forecast}}[m] = [0, 0.01, 0.99].$$

4.3.2 Identifying the optimal decision

For this decision problem, we chose to calculate the number of cases averted, $\hat{C}$, under deployment of each antiviral strategy $d \in \{Rxall/PEPall, Rxall, RxARhosp\}$, compared to *Inaction*. Once we have the posterior probability of `Scenario`, we use it along with the intervention model to calculate the expected number of cases averted $\hat{C}$ under deployment of each antiviral strategy, given the evidence e:

$$\mathbb{E}[\hat{C}, d|e] = \sum_{i,j} \hat{C}(i,j,d)P(i,j|e), \tag{4}$$

where

$$\hat{C}(i,j,d) = C(i,j,Inaction) - C(i,j,d). \tag{5}$$

We also calculate the standard deviation σ for cases averted:

$$\sigma(\hat{C}(i,j,d)) = \sqrt{\sum_{i,j} \hat{C}(i,j,d)^2 P(i,j|e) - \left(\sum_{i,j} \hat{C}(i,j,d)P(i,j|e)\right)^2}. \tag{6}$$

The `decision-maker` then uses this information (the expected number of cases averted and uncertainty) to identify the optimal decision d^*:

$$d^* = \arg\max_d \left(\texttt{decision-maker}\left(\mathbb{E}[\hat{C}, d|e], \sigma(\hat{C}_{i,j})\right)\right). \tag{7}$$

This very general formulation for how the `decision-maker` operates, in that it simply maximises some utility over decision options d, allows the many other factors not captured by the decision model to influence the ultimate choice of antiviral strategy.

5 Results

The expected number of cases averted under each antiviral strategy given no situational evidence, evidence for Pandemic A, and evidence for Pandemic B is shown in Figure 4. It is important for decision-makers to consider the full `Scenario` posterior distribution, not just the expected values, due to possible underlying asymmetries of the posterior. Thus the figure also displays cases averted (values from Equation 5) for each of the pandemic scenarios, underlaid by a density plot of the `Scenario` posterior. This allows us to assess the relative number of cases averted under each combination of antiviral strategy and pandemic scenario, along with the scenario weightings specific to each hypothetical pandemic. The number of cases averted varies

across pandemic scenarios under each of the antiviral strategies. Thus when the `Scenario` posterior is strongly weighted towards a high severity, low transmissibility scenario (as in Pandemic A), the expected number of cases averted is skewed towards higher values. Conversely when the `Scenario` posterior is strongly weighted towards a low severity, high transmissibility scenario (as in Pandemic B), the expected number of cases averted is skewed towards lower values. In the absence of situational evidence (and a uniform prior is used for `Scenario`), there is higher uncertainty around the expected value, since the `Scenario` posterior is also uniform.

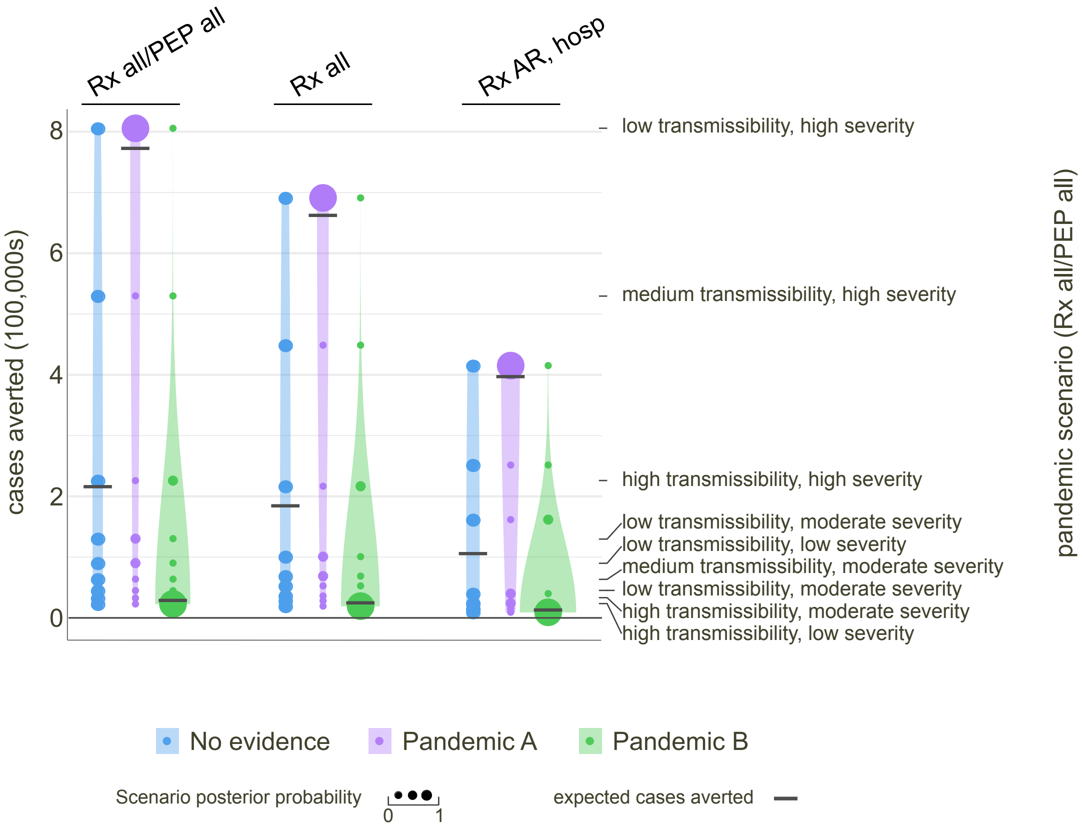

Figure 4: Plots are shown for No evidence, Pandemic A (strong evidence of high severity, low transmissibility scenario) and Pandemic B (strong evidence of low severity, high transmissibility scenario) for each antiviral strategy. The plot for each antiviral strategy and evidence pair shows the cases averted for each pandemic scenario (coloured dots) underlaid by a density plot of the `Scenario` posterior, given the evidence (or the uniform prior, before situational evidence becomes available). The size of each pandemic scenario dot is also proportional to the `Scenario` posterior probability. The expected number of cases averted is indicated by the black bar through each plot. Each of the pandemic scenarios are labelled for antiviral strategy 'Rx all/PEP all', but not the other two strategies.

Thus there are three key results which have implications for decision-making:

1. Uncertainty is greatest before situational evidence becomes available.

2. For all antiviral strategies, the expected cases averted and the underlying posterior distri-

bution are skewed towards higher values when situational evidence suggests a high severity, low transmissibility scenario (Pandemic A) compared to when situational evidence suggests a low severity, high transmissibility scenario (Pandemic B).

3. The expected cases averted is highly variable across antiviral strategies when situational evidence suggests a high severity, low transmissibility scenario (Pandemic A) and not when situational evidence suggests a low severity, high transmissibility scenario (Pandemic B).

Given situational evidence, there are strong grounds to use antivirals, and to use them liberally, in response to Pandemic A. For Pandemic B, there is little evidence to support the widespread use of antivirals as a public health measure. In both cases, antivirals would be used for targeted case treatment.

References

[1] R. Moss, J.M. McCaw, A.C. Cheng, A.C. Hurt, and J. McVernon. Reducing disease burden in an influenza pandemic by targeted delivery of neuraminidase inhibitors: mathematical models in the australian context. *BMC Infectious Diseases*, 16(1):552, 2016.

[2] Australian Government Department of Health. Australian Health Management Plan for Pandemic Influenza. Canberra, August 2014. Available from: `https://www1.health.gov.au/internet/main/publishing.nsf/Content/ohp-ahmppi.htm`.

[3] J.M. McCaw, J.G. Wood, C.T. McCaw, and J. McVernon. Impact of emerging antiviral drug resistance on influenza containment and spread: influence of subclinical infection and strategic use of a stockpile containing one or two drugs. *PLoS ONE*, 3(6):1–10, 06 2008.

[4] J.M. McCaw and J. McVernon. Prophylaxis or treatment? Optimal use of an antiviral stockpile during an influenza pandemic. *Mathematical Biosciences*, 209(2):336 – 360, 2007.

[5] J. McVernon, J.M. McCaw, and T.M. Nolan. Modelling strategic use of the national antiviral stockpile during the CONTAIN and SUSTAIN phases of an Australian pandemic influenza response. *Australian and New Zealand Journal of Public Health*, 34(2):113–119, 2010.

[6] J.M. McCaw, K. Glass, G.N. Mercer, and J. McVernon. Pandemic controllability: a concept to guide a proportionate and flexible operational response to future influenza pandemics. *Journal of Public Health (Oxford, England)*, 36(1):5–12, 2014.

[7] J.M. McCaw, R. Moss, and J. McVernon. A decision support tool for evaluating the impact of a diagnostic capacity and antiviral-delivery constrained intervention strategy on an influenza pandemic. *Influenza and Other Respiratory Viruses*, 5(Suppl. 1):202–229, 2011.

[8] R. Moss, J.M. McCaw, and J. McVernon. Diagnosis and antiviral intervention strategies for mitigating an influenza epidemic. *PLOS ONE*, 6(2):1–10, 02 2011.

[9] K.B. Korb and A.E. Nicholson. *Bayesian Artificial Intelligence, Second Edition.* CRC Press, Inc., Boca Raton, FL USA, 2010.

[10] F.V. Jensen and T.D. Nielsen. *Bayesian Networks and Decision Graphs.* Springer, New York, 2007.

[11] Y. Ye, F. Tsui, M. Wagner, J.U. Espino, and Q. Li. Influenza detection from emergency department reports using natural language processing and Bayesian network classifiers. *Journal of the American Medical Informatics Association*, 21(5):815–823, 2014.

[12] M. Wagner, F. Tsui, G. Cooper, J.U. Espino, H. Harkema, Levander J., R. Villamarin, R. Voorhees, N. Millett, C. Keane, A. Dey, M. Razdan, Y. Hu, M. Tsai, S. Brown, B.Y. Lee, A. Gallagher, and M. Potter. Probabilistic, decision-theoretic disease surveillance and control. *Online Journal of Public Health Informatics*, 3(3), 2011.

[13] A. Beresniak, E. Bertherat, W. Perea, G. Soga, R. Souley, Dupont D., and S. Hugonnet. A Bayesian network approach to the study of historical epidemiological databases: modelling meningitis outbreaks in the Niger. *Bulletin of the World Health Organization*, 90:412–417A, 2012.

[14] P. Dawson, R. Gailis, and A. Meehan. Detecting disease outbreaks using a combined Bayesian network and particle filter approach. *Journal of Theoretical Biology*, 370:171–183, 2015.

[15] R. Moss, J.E. Fielding, L.J. Franklin, N. Stephens, J. McVernon, P. Dawson, and J.M. McCaw. Epidemic forecasts as a tool for public health: interpretation and (re)calibration. *Australian and New Zealand Journal of Public Health*, 42(1):69–76, 2018.

[16] A.J. Black, N. Geard, J.M. McCaw, J. McVernon, and J.V. Ross. Characterising pandemic severity and transmissibility from data collected during first few hundred studies. *Epidemics*, 19:61–73, 2017.

[17] J. Kim and J. Pearl. A computational model for causal and diagnostic reasoning in inference systems. *Proceedings of the Eighth International Joint Conference on Artificial Intelligence (IJCAI)*, pages 190–193, 1983.
